# Measurement of cardiac output in children: comparison between direct Fick method and pressure recording analytical method: preliminary report

**DOI:** 10.1186/cc14250

**Published:** 2015-03-16

**Authors:** J Alonso Iñigo, F Escribá, J Carrasco, J Encarnación, M Fas, M Barberá

**Affiliations:** 1 Hospital Universitario y Politécnico La Fe, Valencia, Spain; 2 Hospital Universitario de la Ribera, Alzira, Spain

## Introduction

There are few methods of cardiac output (CO) estimation validated in children. The aim of this study is to investigate the reliability of an uncalibrated pulse contour method of CO estimation, the pressure recording analytical method (PRAM), in pediatric patients scheduled for diagnostic right and left heart catheterization, compared with the oxygen-direct Fick method.

## Methods

Cardiac index (CI) was simultaneously estimated by Fick, and PRAM applied to pressure signals recorded invasively from a femoral catheter. All measurements were performed in steady-state condition. PRAM CI measurements were obtained for 10 consecutive beats simultaneously during the Fick CI estimation. Agreement between Fick and PRAM was assessed using the Bland-Altman method. Correlation coefficient, bias, and percentage of error were calculated.

## Results

**Figure 1:**
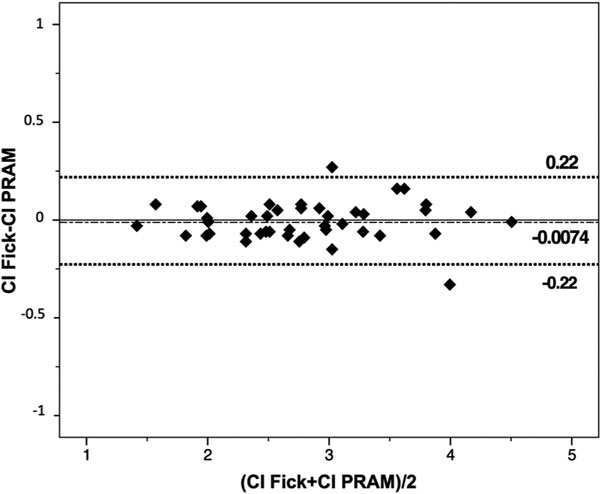
**Bland-Altman plot of the cardiac index measured with Fick versus PRAM**.

Forty-three CI measurements were performed in 43 patients. The data showed good agreement between CIFick and CIPRAM: *r*^2^ = 0.98; bias -0.0074 l/minute/m^2^; limits of agreement from -0.22 to 0.22 l/minute/m^2^. The percentage error was 8%. Figure 1 shows the Bland- Altman plot.

## Conclusion

PRAM provides reliable estimates of cardiac output in hemodynamically stable pediatric cardiac patients compared with the Fick method.
